# A210 ESOPHAGEAL SQUAMOUS PAPILLOMATOSIS AFTER ENDOSCOPIC SUBMUCOSAL DISSECTION

**DOI:** 10.1093/jcag/gwad061.210

**Published:** 2024-02-14

**Authors:** R Gilhotra, A Zarrin, S X Jiang, W Xiong, N Shahidi

**Affiliations:** The University of British Columbia Faculty of Medicine, Vancouver, BC, Canada; The University of British Columbia Faculty of Medicine, Vancouver, BC, Canada; The University of British Columbia Faculty of Medicine, Vancouver, BC, Canada; The University of British Columbia, Vancouver, BC, Canada; The University of British Columbia Faculty of Medicine, Vancouver, BC, Canada

## Abstract

**Background:**

Squamous papillomatosis is a rare benign epithelial finding, generally found incidentally in the distal esophagus. The pathophysiology is not well understood but felt to be secondary to reflux and chemical irritation leading to leukoplakia and verrucous hyperplasia of the epithelium and papilloma formation.

**Aims:**

To present a case of esophageal squamous papillomatosis after endoscopic submucosal dissection (ESD) and radiofrequency ablation (RFA) of Barrett’s esophagus (BE) and early esophageal cancer.

**Methods:**

Case report and review of the literature

**Results:**

A 78-year-old male with C3M3 BE and previous band mucosectomy for nodular dysplasia was referred for ESD of an early esophageal cancer. ESD was successfully performed, with histopathology identifying a low-risk well-differentiated intramucosal adenocarcinoma without lymphovascular invasion and negative margins. Radiofrequency ablation was performed 3-months post-resection for residual BE. Subsequent surveillance gastroscopy 6 months post-resection identified extensive fleshy pink granulation tissue at the ESD scar, histopathology revealing SP. This was confirmed on subsequent repeat gastroscopy and biopsy. Compliance with double dose proton pump inhibitor therapy was confirmed.

**Conclusions:**

To our knowledge, this is the first reported case of SP developing post-ESD and RFA; further expanding our knowledge of potential Barrett’s endotherapy-related adverse events.

**Funding Agencies:**

None

